# Preparation and Properties of Transparent, Thermally Insulating, and Flexible SiO_2_ Aerogels

**DOI:** 10.3390/ma19112401

**Published:** 2026-06-04

**Authors:** Jian Li, Shuhang Shi, Haitao Shu, Qianyu Chen, Yun Zhou, Ying Yuan, Xiaotian Peng

**Affiliations:** 1 Chongqing Anting Environmental Protection Technology Co., Ltd., Chongqing 400064, China; 17368483057@163.com; 2 Jiangsu Key Laboratory of Process Enhancement and New Energy Equipment Technology, School of Mechanical and Power Engineering, Nanjing Tech University, Nanjing 211816, China; 13913535794@163.com (S.S.); 17349800893@163.com (H.S.); 202261207229@njtech.edu.cn (Q.C.); 3 Special Equipment Safety Supervision Inspection Institute of Jiangsu Province, Nanjing 210036, China; jsseiyuany@163.com; 4 Technology Innovation Center of Safety Operations and Maintenance for Oil and Gas Storage and Transportation Equipment Facilities for Jiangsu Province Market Regulation, Nanjing 210036, China

**Keywords:** SiO_2_ aerogel, composite silicon source, transparency, thermal insulation, flexibility

## Abstract

**Highlights:**

- A transparent, flexible SiO_2_ aerogel was prepared via ambient pressure drying using an optimized MTMS–DMDMS composite silicon source.
- The aerogel achieves a visible light transmittance of 89.74% and a low thermal conductivity of 0.027 W/(m·K).
- The introduction of DMDMS enhances flexibility, increasing compressive strength by 22.4% compared to pure MTMS aerogels.
- The material exhibits excellent hydrophobicity with a water contact angle of 125.98° and a low density of 0.1802 g/cm^3^.
- A multi-parameter cooperative regulation strategy enables balanced optimization of optical, thermal, and mechanical properties.

**The main findings.**

- Using tetramethylammonium hydroxide (TMAOH) as the alkaline catalyst, a water/MTMS molar ratio of 7:1, and Pluronic F-127 (F127) as the surfactant yields a single-silicon-source SiO_2_ aerogel with uniform pore structure, 93.50% porosity, 125.98° water contact angle, and 89.74% transmittance at 800 nm.
- Constructing an MTMS–DMDMS composite silicon source with a molar ratio of 9:1, 6 mL water addition, and 2.0 g F127 produces a flexible aerogel with 22.4% higher compressive strength, 70.15% visible transmittance, and thermal conductivity as low as 0.027 W/(m·K), achieving balanced mechanical, optical, and thermal insulation properties.

**The implications of the main findings.**

- The optimized single-silicon-source preparation route enables stable, low-cost ambient pressure synthesis of transparent, hydrophobic SiO_2_ aerogels, supporting scalable manufacturing for building energy-saving applications.
- The MTMS–DMDMS composite system resolves the trade-off between transparency, thermal insulation, and mechanical flexibility, providing a design paradigm for high-performance transparent thermal insulation materials used in glazing, smart facades, and optical insulation components.

**Abstract:**

SiO_2_ aerogels are promising candidates for energy-efficient glazing because of their low thermal conductivity and optical transparency; however, conventional formulations often fail to reconcile optical, thermal, and mechanical performance. This work aimed to resolve this bottleneck via controllable sol–gel synthesis and ambient pressure drying. Using methyltrimethoxysilane (MTMS) as the single silicon source, this study systematically explored the effects of alkaline catalyst type, water-to-MTMS ratio, and surfactant selection, and further developed an MTMS–dimethyl dimethoxy silicane (DMDMS) composite silicon source. Tetramethylammonium hydroxide (TMAOH) catalysis, a water-to-MTMS molar ratio of 7:1, and Pluronic F-127 (F127) surfactant yielded a uniform, hydrophobic aerogel with 93.50% porosity and 89.74% transmittance at 800 nm. The optimized composite system (MTMS:DMDMS = 9:1, 6 mL water, 2.0 g F127) enhanced compressive strength by 22.4% relative to pure MTMS aerogel, with 70.15% visible transmittance and thermal conductivity of 0.027 W/(m·K). These results demonstrate that multi-parameter formulation control can achieve a practical balance among mechanical robustness, optical transparency, and thermal insulation. This study provides a theoretical and process foundation for the engineering application of high-performance transparent thermal insulation materials.

## 1. Introduction

Heat loss through glazing in building envelopes accounts for more than 50% of the total building load due to the high thermal conductivity of glass, making the enhancement of its thermal insulation performance a critical direction for building energy efficiency [1,2]. Aerogel materials, characterized by their ultra-low density, ultra-high porosity, and concurrently excellent thermal and optical properties, have emerged as a promising new option for glass composites [3,4,5,6].

Aerogel glazing has been gradually utilized since 1977 [7]. Leung et al. [8] demonstrated that aerogel glazing could reduce window heat gain by 57%. Rejeb et al. [9] developed molecularly bridged flexible silica aerogels via ambient pressure drying, which exhibited excellent hydrophobicity with a water contact angle up to 143.6°, outstanding compressibility up to 99% strain, and low density of 0.098–0.12 g/cm^3^. The aerogel glass units developed by Abraham et al. [10] and Riichi et al. [11] both attained a visible light transmittance of 95%, significantly improving visual clarity and light scattering performance. Jensen et al. [12] reported that filling glass units with aerogel could reduce building energy consumption by 29% and decrease the U-value by over 50%. Subsequent studies have shown that the U-value of granular aerogel can reach 0.44–1.31 W/(m^2^·K) [13,14], while monolithic aerogel has seen increased application in recent years due to its superior optical properties. For instance, Berardi [13] incorporated a 12 mm monolithic aerogel layer into glass, achieving a U-value as low as 0.6 W/(m^2^·K). Several global patented applications or demonstration projects, such as the aerogel mosaic windows in the United States [15], aerogel glass bricks from the Swiss Federal Laboratories for Materials Science and Technology [16], and projects by the Worcester Polytechnic Institute in the USA [13], have validated its energy-saving and optical performance.

The preparation of aerogel glass primarily employs the sol–gel method, which encompasses four steps: sol–gel processing, aging, solvent exchange, and drying [17]. Among these, the sol–gel process is the key step determining the material’s structure and properties, typically using alkoxysilanes as precursor [18]. Bar et al. [19] fabricated silica aerogel films via tetraethyl orthosilicate (TEOS), achieving a transmittance of 86% at 1100 nm; however, these films suffered from drawbacks such as high cost, brittleness, and strong hydrophilicity [20]. To overcome these issues, methyl-capped precursors like methyltrimethoxysilane (MTMS) and methyltriethoxysilane (MTES) have been widely adopted [21,22]. Niu et al. [23] prepared aerogels using MTES with a thermal conductivity of 0.048–0.052 W/(m·K), but the visible light transmittance was only 65–69%. Sun et al. [24] obtained aerogels with a thermal conductivity as low as 0.0237 W/(m·K) through a modified process, yet the transmittance remained insufficient. Shi et al. [25] prepared aerogels via supercritical drying using tetramethyl orthosilicate (TMOS), achieving a transmittance of 97.78% at 800 nm; however, challenges such as long preparation cycles, high equipment requirements, and poor mechanical properties persisted. In summary, although single-silicon-source aerogels can have their optical transparency enhanced through modification, they still face challenges including immature preparation processes, difficulty in balancing properties, and insufficient long-term stability [26].

To enhance mechanical properties and stability, multi-silicon-source composite systems have gradually become a research hotspot [27]. Wen et al. [28] prepared a SiO_2_-Al_2_O_3_ composite aerogel, which significantly improved the skeleton structure and thermal stability. Zhang et al. [29] developed a silylated cellulose aerogel that maintained a thermal conductivity as low as 0.0159 W/(m·K) even under high-humidity conditions. Ren et al. [30] fabricated an aerogel by compositing MTES with polyhedral oligomeric silsesquioxane (POSS), achieving excellent compressive resilience up to 70% strain and superior fatigue resistance (30 cycles at 40% strain). Although composite silicon sources can enhance thermodynamic performance, environmental adaptability, and confer functionalization potential, achieving a balance among optical transparency, thermal insulation, and mechanical strength remains a major challenge. Overall, aerogel glass is widely recognized as a promising approach for reducing energy consumption, offering advantages in thermal insulation, mechanical stability, and hydrophobicity. However, transparent aerogel glazing still faces challenges during its preparation and modification, including poor mechanical properties of the gel, low transparency, insufficient thermal stability, high production costs, and complex processing, which need to be resolved [7].

To address these challenges, this study focused on the preparation of aerogels with low density, low thermal conductivity, and high transparency. Initially, for the ambient pressure preparation of single-silicon-source aerogels, MTMS was selected as the precursor. Different types and ratios of alkaline catalysts were designed to investigate their effects on gelation time and transparency. The influence of formulation ratios and surfactant types on the microstructure, thermal properties, hydrophobicity, and preparation cycle of SiO_2_ aerogels was systematically studied. Building on this, a multi-silicon-source composite strategy employing MTMS and DMDMS was adopted. By regulating the condensation rates and cross-linking degrees of different silicon sources, the synergistic optimization of mechanical, thermal, and optical properties was achieved. The novelty of this work lies in three aspects. First, an MTMS–DMDMS composite precursor system was designed to mitigate the trade-off between optical transparency and mechanical flexibility. Second, a facile ambient pressure drying route was adopted, avoiding the need for supercritical drying equipment. Third, multi-parameter formulation control was used to regulate the pore structure and improve the overall balance among optical, thermal, and mechanical properties. The ultimate goal was to obtain aerogel materials with structural integrity, high transparency, excellent hydrophobicity, and a short preparation cycle, thereby providing experimental support and process references for engineering applications in the field of transparent thermal insulation.

## 2. Materials and Methods

### 2.1. Materials and Instruments

The main chemical reagents involved in the aerogel synthesis and characterization, along with their purity and manufacturers, are listed in Table 1.

The instruments and equipment involved in the experiment are shown in Table 2.

### 2.2. Experimental Procedure

**Figure 1:**
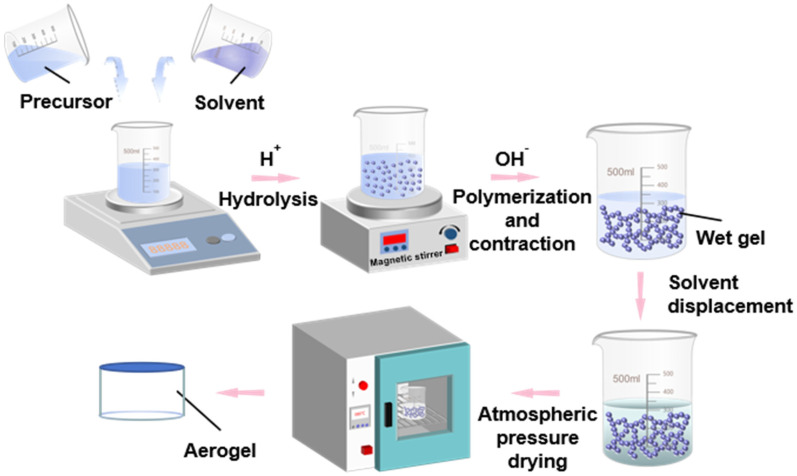
Aerogel preparation process.

MTMS-based SiO_2_ aerogels were prepared by an acid-base two-step catalyzed sol–gel process followed by ambient pressure drying. MTMS was used as the precursor, CTAB or F127 as the surfactant, deionized water as the co-solvent, and acetic acid along with aqueous ammonia/Urea/TMAOH as the acid and base catalysts, respectively. The specific process flowchart is shown in Figure 1 and comprises the following four key steps:

(1) Sol–Gel Process: Deionized water, acetic acid, and MTMS were mixed at a set molar ratio and stirred uniformly at room temperature. The mixture was then allowed to hydrolyze under ambient conditions for 30 min to obtain the sol. Subsequently, an aqueous solution of the base catalyst was gradually added to the hydrolyzed solution to adjust the pH value. After stirring, the mixture was transferred to a temperature-controlled environment for gelation.

(2) Aging: The wet gel, after complete hydrolysis and polycondensation, was demolded and immersed in anhydrous ethanol for aging. The gel remained fully submerged in the ethanol for 24 h, during which the ethanol was replaced twice.

(3) Solvent Exchange: The wet gel was then immersed in n-hexane for solvent exchange over 24 h, with the n-hexane being replaced twice to ensure complete replacement of water within the gel.

(4) Drying: Finally, the wet gel after solvent exchange was placed in a drying oven. Given the ambient pressure drying conditions, a multi-stage drying protocol was employed: primary drying at 60 °C for 6 h, secondary drying at 100 °C for 4 h, and tertiary drying at 20 °C for 4 h.

In this study, the test was conducted in three repeated experiments, and the data were presented in the form of averages.

## 3. Results and Analysis of Single-Silicon-Source Aerogels

### 3.1. Effect of Type and Dosage of Alkaline Catalyst on Gel State

In the preparation of SiO_2_ aerogels, alkaline catalysts play a decisive role in the polycondensation reaction and the gel network structure by regulating the pH of the sol–gel system. When the concentration of the alkaline catalyst was below a critical value, the gelation time increased significantly, and the network cross-linking was insufficient. Conversely, an excessive amount of catalyst led to inhomogeneous gelation. In this study, with the molar ratio of the silicon source, water, and acetic acid fixed at *n*(MTMS:H_2_O:CH_3_COOH) = 1:7:3 and the acetic acid concentration at 5 mmol/L, three alkaline catalysts—aqueous ammonia, urea, and TMAOH—were selected. Then, the effects of their type and dosage on the gel characteristics were systematically investigated.

**Figure 2:**
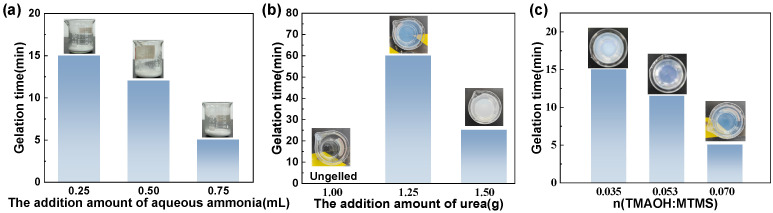
(**a**) Effect of ammonia addition on gel time. (**b**) Effect of urea addition on gel time. (**c**) Effect of *n*(TMAOH:MTMS) on gel time.

When aqueous ammonia was used as the catalyst, as shown in Figure 2a, within the addition range of 0.25–0.75 mL, the system pH increased exponentially with the dosage, and the gelation time decreased from 15 min to 5 min. However, when the dosage exceeded 0.5 mL, the excessively high pH value caused the polycondensation reaction to proceed too rapidly. The gel exhibited a milky white, turbid state, where enhanced Rayleigh scattering led to a decrease in transmittance and potentially induced stress concentration, adversely affecting material stability. This indicates that its use at high concentrations is not suitable for the preparation of highly transparent aerogels.

The results using urea as the alkaline catalyst are shown in Figure 2b. The results show that with an addition of 1.0 g, the sample did not gel even after being maintained at 60 °C for 24 h. At 1.25 g, the gelation time was 60 min with moderate transparency, while at 1.5 g, the gelation time shortened to 25 min but the transparency decreased significantly. The experiment revealed that the urea decomposition process was difficult to control precisely, presenting drawbacks such as long and highly variable gelation times, making it challenging to meet process controllability requirements.

The results for the system using TMAOH are shown in Figure 2c. Within the range of *n*(TMAOH:MTMS) = 0.035:1 to 0.071:1, the gelation time decreased from 15 min to 5 min. Moreover, the rapid increase in system pH preferentially promoted the hydrolysis reaction, leading to the formation of a uniform network structure. When the dosage was further increased, the excessively high system viscosity affected the stirring efficiency. This indicates that a low dosage of TMAOH is sufficient to establish a mild, homogeneous and effective alkaline environment and inhibit phase separation. This regulated reaction kinetics contributes to the formation of a continuous, uniform silica network with minimal light scattering centers.

Comprehensively comparing the reaction efficiency, structural homogeneity, and optical performance of the three catalysts, this study identified TMAOH as the optimal choice. Its optimal addition amount was determined to be *n*(TMAOH:MTMS) = 0.071:1, enabling efficient and uniform aerogel preparation.

### 3.2. Effect of Water-to-MTMS Molar Ratio on Aerogel Properties

During the hydrolysis reaction, MTMS reacted with water under acid catalysis to form silanolates and methanol. In the polycondensation stage, CH_3_-Si-(OH)_3_ formed the Si-O-Si backbone structure under the action of the alkaline catalyst. Both reactions were reversible. Water acted not only as a reactant in hydrolysis and a product in polycondensation but also as the solvent medium. Therefore, the amount of water not only regulates the rates of the hydrolysis and polycondensation reactions of MTMS but also directly affects the construction and stability of the gel network. When the molar ratio of H_2_O to MTMS was high, it promoted the hydrolysis reaction, generating a large number of silanolates and resulting in a loose gel network structure, manifested as a significant increase in porosity. Under conditions of a lower molar ratio, the hydrolysis reaction was inhibited, polycondensation becomes dominant, and the resulting aerogel structure was dense with low porosity. Both excess and insufficient water induce variations in the aerogel’s physical properties. In this experiment, with *n*(TMAOH:MTMS) fixed at 0.071:1 and process parameters such as aging and solvent exchange steps kept constant, molar ratios of *n*(H_2_O:MTMS) = 6:1, 7:1, 8:1, and 9:1 were selected for experimental investigation.

(1) Gelation Time, Density, and Porosity

The apparent density of the material was calculated by the mass-to-volume ratio. The porosity was calculated using Equation (1), where the skeletal density of silica (*ρ*_1_) was set as 2.2 g/cm^3^.

(1) $$P=\left(1-\frac{\rho }{\rho_{1}}\right)\times 100\%$$

**Figure 3:**
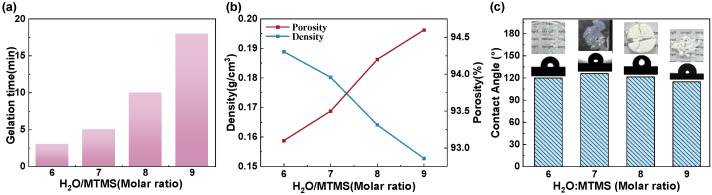
(**a**) Effect of *n*(H_2_O:MTMS) on gelation time. (**b**) Effect of *n*(H_2_O:MTMS) on density, and porosity. (**c**) Effect of *n*(H_2_O:MTMS) on the hydrophobicity of aerogels.

The effects of different *n*(H_2_O:MTMS) ratios on the gelation time and the density and porosity of the aerogels are shown in Figure 3a. As the water-to-MTMS ratio increased from 6:1 to 9:1, the gelation time prolonged from 4 min to 18 min. This phenomenon originated from the decreased intensity of the acidic environment due to the increased water addition, which subsequently slowed the hydrolysis reaction rate. The sparser distribution of hydrolysis products in the solvent reduced the collision probability between Si-OH groups, thereby inhibiting the formation of Si-O-Si linkages during polycondensation. This process lowered the silicon content and delayed the gelation process. Concurrently, it led to a decrease in the compactness of the gel skeleton, manifested as a reduction in aerogel density with increasing water-to-MTMS ratio. Furthermore, as the *n*(H_2_O:MTMS) ratio increased, the slowed polycondensation reaction resulted in a decreased cross-linking degree of the network structure, causing the pore structure to become looser (Figure 3b) and ultimately leading to increased porosity. At a water-to-MTMS ratio of *n*(H_2_O:MTMS) = 7:1, the prepared aerogel exhibited a density of 0.1802 g/cm^3^ and a porosity of 93.50%.

(2) Macroscopic Morphology and Hydrophobicity

The cracking phenomenon of aerogels during the drying process was primarily influenced by both the gel network structure and capillary forces. The effects of different *n*(H_2_O:MTMS) ratios on the macroscopic morphology of the aerogels are shown in Figure 3c, where all samples exhibited distinct cracking characteristics. At *n*(H_2_O:MTMS) = 6:1, the low water content led to incomplete hydrolysis of MTMS and a relatively high concentration of silicic acid monomers, which significantly accelerated the polycondensation rate, forming a dense gel network with high cross-linking. This structure generated intense contraction stress due to capillary forces during drying, ultimately causing skeleton fragmentation. Under the condition of *n*(H_2_O:MTMS) = 7:1, an appropriate amount of water promoted sufficient hydrolysis of MTMS, achieving a dynamic balance between hydrolysis and polycondensation rates and constructing a uniform and stable gel network. This structure effectively resisted capillary forces during drying, significantly reducing volume shrinkage and the degree of cracking. When the *n*(H_2_O:MTMS) ratio increased to 8:1 and 9:1, the excess water accelerated the hydrolysis reaction, while polycondensation lagged relatively, resulting in a loose, fragile, and heterogeneous gel network. The insufficient cross-linking of this structure could not withstand the contraction stress during drying, leading to eventual skeleton collapse and fragmentation. Furthermore, due to the decreased skeleton density and increased porosity, multiple light scattering within the aerogel was intensified, causing a significant decrease in sample transparency.

The hydrophobicity of SiO_2_ aerogels originates from the hydrophobic layer formed by methyl groups on the skeleton surface, which inhibits the adsorption of water molecules. As shown in Figure 3c, when *n*(H_2_O:MTMS) = 6:1, insufficient hydrolysis resulted in an uneven distribution of methyl groups and a relatively high content of surface hydroxyl groups (-OH), leading to decreased hydrophobicity. At *n*(H_2_O:MTMS) = 7:1, the synergistic effect of hydrolysis and polycondensation enabled uniform distribution of methyl groups, forming a stable hydrophobic layer. At this point, the sample exhibited the highest water contact angle, with three measured values of 125.50°, 126.44°, and 126.00°, giving an average value of 125.98°. This result indicates optimal hydrophobicity under this formulation. When the *n*(H_2_O:MTMS) ratio increased to 8:1 and 9:1, insufficient polycondensation caused an imbalanced distribution of methyl groups and a loose skeleton structure, weakening hydrophobicity and resulting in a decreased contact angle.

In summary, at *n*(H_2_O:MTMS) = 7:1, the hydrolysis and polycondensation rates of the sol–gel reaction achieved an optimal balance. The resulting gel network was uniform and stable, leading to aerogels with intact macroscopic morphology, a gelation time of 5 min, and exhibiting the lowest density (0.1802 g/cm^3^), the highest porosity (93.50%), and the best hydrophobicity (contact angle of 125.98°). Therefore, this study identified *n*(H_2_O:MTMS) = 7:1 as the optimal experimental ratio. These results further suggest that an appropriate water dosage ensures sufficient hydrolysis of Si-OCH_3_ groups and a moderate polycondensation rate, thereby forming a robust yet flexible gel network capable of resisting capillary stress during drying. Excess water dilutes the silicate species and leads to loose cross-linking, whereas insufficient water causes incomplete hydrolysis and dense, brittle networks.

### 3.3. Effect of Surfactant Type on Aerogel Properties

Under ambient pressure drying conditions, the structural integrity and optical properties of aerogels can be effectively enhanced through network structure reinforcement and surface modification. In this study, using CTAB and F127 as surfactants, and based on the formulation system with *n*(H_2_O:MTMS) = 7:1 (TMAOH addition amount: *n*(TMAOH:MTMS) = 0.071:1), highly transparent monolithic SiO_2_ aerogels were prepared via the ambient pressure drying process.

(1) Gelation Time, Density, and Porosity

Under ambient pressure drying conditions, the aerogel prepared using CTAB as the surfactant exhibited surface cracks and poor transparency. The mechanism lies in the fact that while CTAB could partially inhibit phase separation during the gelation process, it was difficult to effectively alleviate the shrinkage and cracking caused by capillary forces in the drying stage. In contrast, when F127 was employed as the surfactant, the aerogel displayed an intact overall structure without cracks and a significantly improved transparency. This indicates that F127 possesses a stronger ability to regulate phase separation within the gel system, which can optimize the network structure and simultaneously enhance both the macroscopic morphology and optical properties.

**Figure 4:**
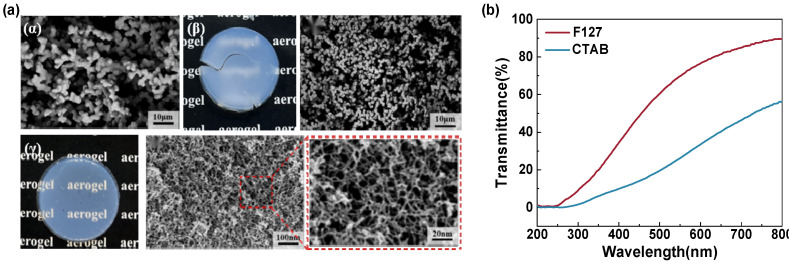
(**a**) Microscopic structure of aerogels: (α) Without additive, (β) CTAB, (γ) F127. (**b**) Schematic diagram of aerogel transmittance.

The macroscopic morphology and microstructure of the aerogels are shown in Figure 4a, revealing the regulatory mechanism of surfactants on the skeleton structure: Figure 4a (α) shows the sample without surfactant addition, presenting a typical “pearl-necklace-like” skeleton structure formed by the interconnection of spherical particles, with large and unevenly distributed pores. This characteristic originated from the severe phase separation induced by the imbalanced hydrolysis and polycondensation rates of MTMS in polar solvents, resulting in a loose porous network and limited mechanical and optical properties. Figure 4a (β) shows the sample with 0.3 g of CTAB addition, where the porous skeleton formed by particle aggregation was finer, and the uniformity of the pore size distribution was improved. Figure 4a (γ) shows the sample with 0.5 g of F127 addition, exhibiting a denser and continuous “fibrous” three-dimensional network structure, featuring a delicate skeleton with uniformly and densely distributed pores, as well as significant branching and cross-linking nodes.

(2) Light Transmittance

Figure 4b shows the visible light transmittance curves of SiO_2_ aerogels prepared with F127 and CTAB, indicating that both exhibit a trend of gradually increasing and stabilizing with increasing wavelength—namely, lower transmittance in the short-wavelength region and higher transmittance in the long-wavelength region. This phenomenon is attributed to Rayleigh scattering. Specifically, when CTAB was used, the average transmittance across the visible light spectrum (380–780 nm) was 30%, with transmittance exceeding 35% in the 600–800 nm range and reaching a maximum of 56% at 800 nm. In contrast, the F127 system exhibited transmittance exceeding 70% across the same wavelength range, with transmittance above 80% in the 630–800 nm interval and a peak value of 89.74% at 800 nm. Overall, the optical transparency of the F127 system was significantly enhanced compared to the CTAB system. From the perspective of structural regulation, F127 acts as a structure-directing agent that stabilizes colloidal silica particles and inhibits uncontrolled aggregation. Its amphiphilic structure induces the formation of a fibrous, interconnected skeleton rather than a discrete pearl-necklace structure, thereby improving transparency and mechanical integrity. This directly corresponds to its advantages in macroscopic morphological integrity and optical performance. Therefore, F127 was selected as the preferred surfactant for subsequent experimental research.

## 4. Results and Analysis of Composite Silicon Source Aerogel

Building upon the ambient pressure drying preparation of SiO_2_ aerogels from a single silicon source, the introduction of a silicon source bearing flexible organic groups effectively improves mechanical properties and flexibility of SiO_2_ aerogels, significantly enhancing their mechanical performance.

### 4.1. Effect of Initial Silane Ratio on the Properties of MTMS–DMDMS Aerogels

To investigate the influence of different silane ratios on aerogel properties, aerogels with varying silicon source ratios were prepared under fixed conditions: a water addition of 5 mL, *n*(TMAOH:silicon source) = 0.071:1, and an F127 addition of 2.0 g. It should be noted that the addition amount of F127 was temporarily set for the purpose of the experiment and was not the preferred result.

#### 4.1.1. Macroscopic Morphology

**Figure 5:**
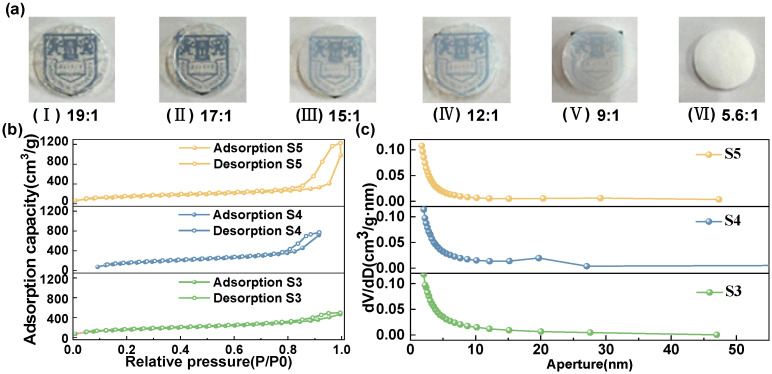
(**a**) Aerogels prepared with different MTMS/DMDMS volume ratios. (**b**) N_2_ adsorption–desorption isotherms of aerogel samples S3–S5. (**c**) Pore size distribution of aerogel samples S3–S5.

Figure 5a shows the appearance of SiO_2_ aerogels prepared with different silane volume ratios, where samples (I) to (VI) correspond to S1 through S6, with *n*(MTMS:DMDMS) ratios of 19:1, 17:1, 15:1, 12:1, 9:1, and 5.6:1, respectively. When the DMDMS proportion was low (S1, S2), the samples appeared as highly transparent monoliths with excellent optical properties. However, due to the relatively rigid siloxane skeleton, significant volume shrinkage occurred during drying, and accumulated internal stress induces crack formation. As the DMDMS proportion increased to 15:1 and 12:1 (S3, S4), the transparency decreased, and the samples exhibited a milky white color, indicating enhanced internal light scattering. When the ratio reached 9:1 (S5), the moderate introduction of dimethyl groups increased the skeleton flexibility, effectively alleviating the drying shrinkage stress. The sample appeared semi-transparent with intact macroscopic morphology. When the DMDMS proportion reached 5.6:1 (S6), the excessive skeleton flexibility reduced the structural compactness, significantly enhanced light scattering, and resulted in a completely opaque, milky white sample.

As shown in Figure 5a, samples S1 and S2 both developed cracks after drying, exhibiting unsatisfactory mechanical properties. In contrast, sample S6 appeared milky white and completely opaque, failing to meet the optical performance requirements of this study. Comparatively, samples S3, S4, and S5 achieved a good balance between mechanical strength and transparency and were therefore identified as the primary subjects for subsequent detailed analysis. The data in Table 3 show that as the DMDMS ratio increased from 15:1 to 9:1 (S3 to S5), the specific surface area displayed a slight decreasing trend, while the pore volume and average pore diameter increased significantly. This phenomenon indicated that the introduction of DMDMS played a significant regulatory role in the siloxane network structure, achieving an expansion of pore volume by reducing the skeletal density.

The nitrogen adsorption–desorption isotherms of samples S3–S5 are shown in Figure 5b. All samples exhibited characteristics of Type IV isotherms. In the low relative pressure region (P/P_0_ < 0.2), the adsorption quantity was low, indicating a low content of micropores and suggesting that the pore structure was dominated by mesopores and macropores. Within the medium relative pressure range (0.2 < P/P_0_ < 0.8), the adsorption quantity increased significantly, corresponding to the capillary condensation effect in mesopores. The adsorption quantity of S5 was much higher than that of S3, indicating that a higher DMDMS ratio led to a looser skeleton, providing a larger pore volume. The presence of a hysteresis loop at high relative pressure (P/P_0_ > 0.9) confirmed that the material was primarily mesoporous. Furthermore, as the DMDMS ratio increased, the range of the hysteresis loop gradually expanded, reflecting an increase in pore volume and an evolution of the pore structure towards a mesoporous system with larger pores. This trend is consistent with the data in Table 3.

The pore size distribution curves in Figure 5c show that the pores of S3 were concentrated in the 6–10 nm range with a narrow distribution, indicating a high degree of cross-linking in its siloxane network and a uniform, dense pore structure. For sample S4, the pore size distribution broadened to 9–20 nm, as DMDMS weakened the network cross-linking, leading to enlarged pores. The pore size of S5 further extended to 20–50 nm. The concentrated distribution of large pores matched its high pore volume value of 3.31 cm^3^/g, confirming that DMDMS acts as a “diluent” in the siloxane network, promoting the formation of macropores by reducing the skeletal compactness.

#### 4.1.2. Optical Characterization

**Figure 6:**
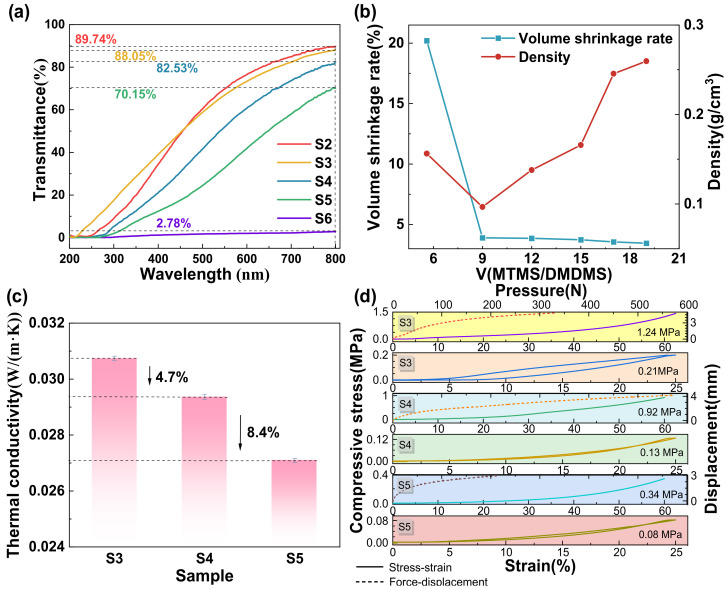
(**a**) Visible light transmittance of aerogels with different MTMS/DMDMS volume ratios. (**b**) Volume shrinkage ratio and density of aerogels. (**c**) Thermal conductivity of aerogel samples S3–S5. (**d**) Stress–strain and compression resilience curves of aerogel samples S3–S5.

Figure 6a shows the curves of visible light transmittance versus wavelength for samples S2 to S6. Due to its low MTMS proportion, sample S6 exhibited a visible light transmittance of only 2.78%. As the MTMS proportion increased (samples S5, S4), the cross-linking density of the siloxane network was enhanced, the skeleton structure became more compact, and the pore size distribution became more uniform. This led to a significant weakening of the light scattering effect, and the visible light transmittance increased to 70.15% and 82.53%, respectively. When the MTMS ratio was further increased to 17:1 (sample S2) and 15:1 (sample S3), the visible light transmittance reached 88.05% and 89.74%, respectively, approaching the theoretical limit. This indicates that the cross-linking degree of the siloxane network approached saturation, resulting in a stable and uniform skeleton structure that minimized light scattering. The above results demonstrate that the pore structure and optical properties of the aerogels can be effectively regulated by optimizing the MTMS/DMDMS ratio. Within the MTMS/DMDMS molar ratio range of 9:1 to 17:1 (samples S2–S5), the aerogels exhibited transmittance exceeding 70% at a wavelength of 800 nm, demonstrating their potential for application in the field of transparent thermal insulation materials.

Figure 6b shows the trends of volume shrinkage and density of aerogel samples S1–S6 with varying composite silicon source ratios. As the MTMS/DMDMS ratio increased, the volume shrinkage decreased significantly and stabilized. Sample S6 exhibited a volume shrinkage of approximately 20%, indicating that its network skeleton possessed relatively weak rigidity, leading to deformation and shrinkage due to internal stress during drying. Furthermore, although the introduction of dimethyl groups reduced the material’s brittleness, it resulted in a higher shrinkage rate. When the ratio increased to 9:1 (samples S4, S5), the enhanced rigidity of the silicon skeleton caused the volume shrinkage to decrease rapidly to a stable state. The shrinkage rate of samples S1–S3 further decreased to about 2%, demonstrating an extremely low shrinkage characteristic.

The density curve also reveals that the aerogel density increased with the rising MTMS/DMDMS ratio, reflecting the enhanced cross-linking density of the siloxane network and the densification process of the skeleton. Due to its high volume shrinkage, sample S6 had a relatively high density of approximately 0.16 g/cm^3^. The large number of dimethyl groups introduced by the high DMDMS content reduced the cross-linking density, allowing the skeleton to maintain a certain degree of looseness. Samples S4 and S5, with their higher DMDMS proportions, had loose skeletons and high porosity, resulting in densities of 0.14 g/cm^3^ and 0.09 g/cm^3^, respectively. For samples S1–S3, the increased MTMS proportion significantly enhanced the cross-linking density of siloxane bonds, leading to the gradual densification of the skeleton structure and a notable improvement in the uniformity of the pore distribution.

#### 4.1.3. Thermal and Mechanical Characterization

Figure 6c shows the variation trend of the thermal conductivity of samples S3, S4, and S5 with the composite silicon source ratio. The experiments demonstrated that as the proportion of DMDMS increased, the thermal conductivity of the aerogels exhibited a decreasing trend. For instance, sample S3 had a thermal conductivity of 0.030 W/(m·K). When the DMDMS ratio was increased to 12:1 (S4) and 9:1 (S5), the thermal conductivity decreased to 0.0287 W/(m·K) and 0.0273 W/(m·K), respectively, corresponding to reductions of 4.7% and 8.4%. This phenomenon originated from the large number of organic groups (e.g., -CH_3_) introduced by DMDMS, which increased the porosity and reduced the pathways for solid-phase heat conduction.

The results indicated that optimizing the thermal performance could be achieved by adjusting the MTMS/DMDMS ratio while maintaining a synergistic balance between the pore structure and mechanical properties. Among them, samples S4 and S5 with higher DMDMS proportions (*n* = 12:1 and 9:1) showed significant advantages in terms of thermal conductivity, validating the inhibitory effect of the organic-inorganic composite structure on heat conduction.

Figure 6d shows the compressive strain–stress curves and resilience characteristics of aerogel samples S3–S5. The experiments demonstrated that sample S3 exhibited the highest compressive strength, reaching 1.24 MPa, while those of S4 and S5 decreased to 0.92 MPa and 0.34 MPa, respectively, showing a decreasing trend with increasing DMDMS proportion. It is noteworthy that none of the samples exhibited brittle unloading characteristics, indicating excellent material flexibility.

In the force–displacement relationship, the curve for S3 was steep, reflecting its higher rigidity during the initial compression stage and outstanding skeleton resistance to deformation. The curves for S4 and S5 showed a significantly increased degree of bending, with S5 in particular demonstrating excellent compressibility and a markedly greater displacement compared to the other samples. This deformability originated from the flexible skeleton imparted by the high DMDMS proportion, enabling it to maintain structural integrity under substantial compressive deformation and possess adaptability to dynamic loads. A synergistic balance between compressive strength and flexibility can be achieved by adjusting the MTMS/DMDMS volume ratio. Samples with a high MTMS proportion (e.g., S3) exhibited superior compressive performance, while those with a high DMDMS proportion (e.g., S5) possessed better deformation recovery capability.

#### 4.1.4. Summary

**Figure 7:**
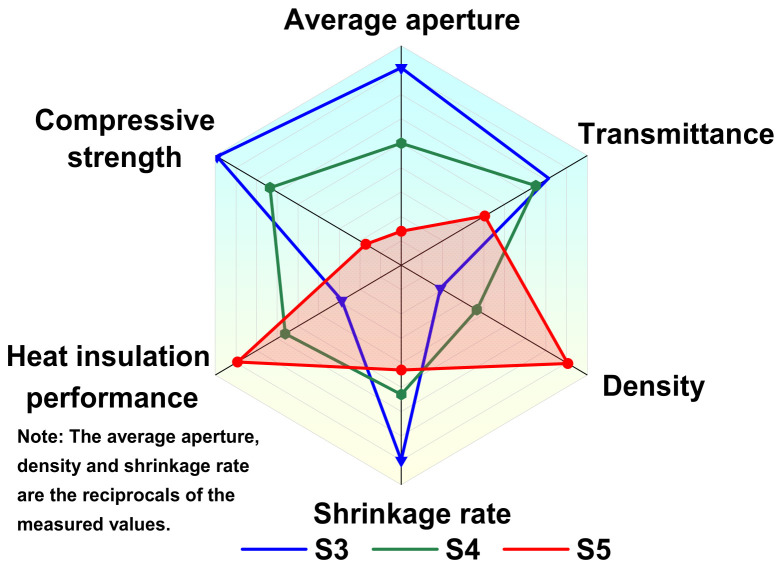
Comparison of the characterization parameters of samples S3–S5, normalized by the maximum value of each characteristic.

Figure 7 compares the various parameters of aerogel samples with different silicon source ratios. Among them, the red polygon representing sample S5 indicates that it performs the best in terms of density and thermal insulation properties. Although its light transmission performance is the poorest, its transmittance still exceeds 70%, which has practical application value. Moreover, its compressive resistance and other properties can be improved in subsequent research. Considering that sample S5 had the lowest density and the best thermal insulation performance, the optimal silicon source ratio of *n*(MTMS:DMDMS) = 9:1 was selected, providing a foundation for the subsequent research.

### 4.2. Effect of Water Addition Amount on the Properties of MTMS–DMDMS Aerogels

Water, as a key reactant in the sol–gel reaction, played a decisive role in the hydrolysis and polycondensation processes of the siloxane network. Building upon the research on the composite silicon source ratio, with *n*(MTMS:DMDMS) fixed at 9:1 and the F127 addition amount at 2.0 g, the effect of water addition amount on aerogel properties was further investigated.

#### 4.2.1. Macroscopic Morphology

**Figure 8:**
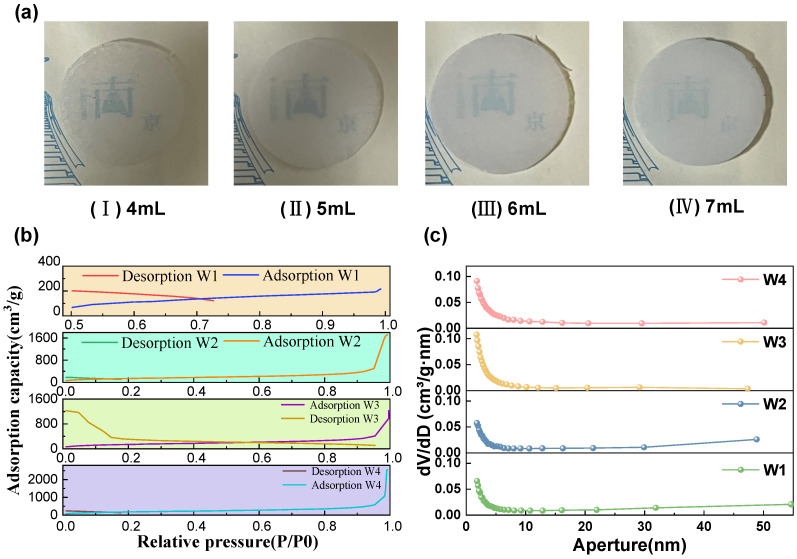
(**a**) Aerogels with different water additions. (**b**) N_2_ adsorption–desorption isotherms of aerogel samples W1–W4. (**c**) Pore size distribution of aerogel samples W1–W4.

Figure 8a shows the appearance of SiO_2_ aerogel samples prepared under different water addition amounts, where (I)–(IV) correspond to samples W1, W2, W3, and W4, with water addition amounts of 4 mL, 5 mL, 6 mL, and 7 mL, respectively.

As the water addition amount increased, the transparency of the aerogels gradually decreased. For instance, W1, due to a balanced hydrolysis–polycondensation reaction, formed a dense and uniform skeleton with high cross-linking density, significantly suppressing the Rayleigh scattering effect and thus exhibiting excellent optical properties. When the water addition amount was increased to 7 mL (W4), the transmittance dropped to its lowest level, nearly completely losing optical transparency. In terms of macroscopic morphology, although samples W1–W3 exhibited gradient differences in transmittance, they all met the basic requirements for optical performance in daily applications. Therefore, after excluding W4 with the lowest transmittance, samples W1–W3 within the water addition range of 4–6 mL possessed both excellent optical properties and process tunability, providing an effective parameter range for subsequent performance optimization.

Table 4 presents the physical properties of the samples. It can be seen that from W1 to W4, the specific surface area, pore volume, and average pore diameter of the aerogels all showed an increasing trend. This indicates that the increase in water addition amount promotes the development of the pore structure, leading to a simultaneous enhancement of specific surface area and pore volume, accompanied by an expansion of the average pore diameter.

Figure 8b shows the nitrogen adsorption–desorption isotherms of samples W1–W4. From the curve morphology, all samples exhibited characteristic Type IV adsorption isotherms. Namely, in the low relative pressure region (P/P_0_ < 0.2), monolayer adsorption occurred with a low adsorption quantity, indicating that the materials contained a minimal amount of micropores, with a limited proportion of micropores. Within the medium relative pressure range (0.2 < P/P_0_ < 0.8), the adsorption quantity increased significantly, corresponding to the capillary condensation effect in mesopores. The presence of a hysteresis loop in the high relative pressure region (P/P_0_ > 0.9) confirmed that the materials were primarily mesoporous (pore size range of 2–50 nm). It is noteworthy that the hysteresis loop range gradually expanded from samples W1 to W4, reflecting an increase in pore volume with increasing water addition and an evolution of the pore structure towards a mesoporous system.

The pore size distribution curves in Figure 8c show that the pores of W1 were concentrated below 10 nm with a narrow distribution, reflecting a dense network structure and small-pore characteristics. The pore size distributions of W2 and W3 shifted towards larger pores with a broadened range, indicating an increased proportion of macropores after regulation. The pore size distribution of W4 extended to the 10–50 nm range with a significantly enhanced peak intensity, suggesting a more developed pore structure with the highest macropore content.

In summary, although nanopores were beneficial for increasing specific surface area and enhancing thermal insulation/adsorption performance, they might compromise mechanical properties. An increase in pore volume enhances the void space within the aerogel, which was crucial for thermal insulation performance. The average pore size directly affected the balance between thermal and mechanical properties. Among the samples, W3 combined a relatively high specific surface area with a large pore volume and a moderate pore size distribution, achieving optimal coordination between thermal insulation performance and mechanical properties.

#### 4.2.2. Optical Characterization

**Figure 9:**
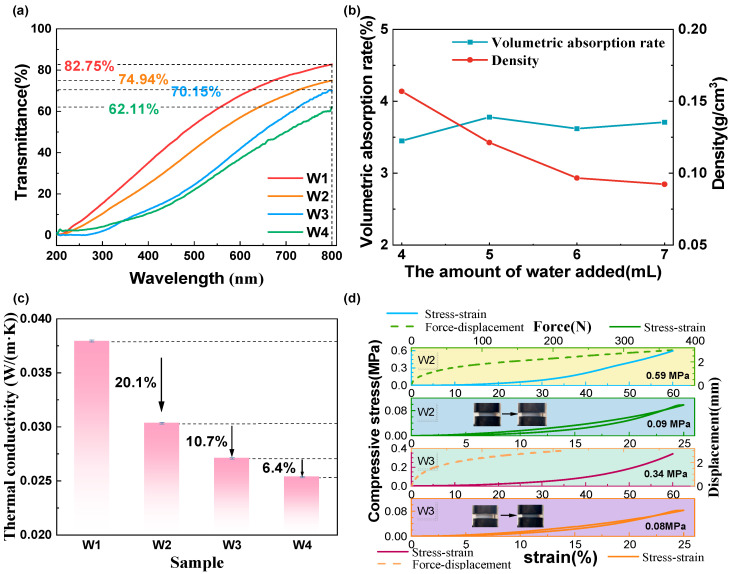
(**a**) Visible light transmittance of aerogels samples W1–W4. (**b**) Volume shrinkage ratio and density of aerogels samples W1–W4. (**c**) Thermal conductivity of aerogel samples W1–W4. (**d**) Stress–strain and compression resilience curves of aerogel samples W2 and W3.

Figure 9a shows the transmittance curves of samples W1–W4 within the visible light range. As the water addition amount increased from 4 mL to 7 mL (corresponding to W1 to W4), the visible light transmittance of the aerogels showed a significant decreasing trend. At a wavelength of 800 nm, the transmittance values of the samples were 82.75%, 74.94%, 70.15%, and 62.11%, respectively.

Samples W1 and W2 exhibited relatively high transparency due to their dense and uniform silicon skeleton structure and reasonable pore distribution, which effectively suppressed the Rayleigh scattering effect. When the water addition amount was increased to 6 mL (W3), the transmittance decreased to 70.15%, indicating that although a certain skeleton density was maintained, the increased light scattering path led to a decline in optical transparency. Upon further increasing the water addition to 7 mL (W4), the transmittance continued to drop to 62.11%. The excessive water caused inhomogeneity in the siloxane network structure, enhancing the scattering effect and thereby significantly compromising the optical performance.

Figure 9b shows the variation patterns of volume shrinkage and density for aerogel samples under different water addition amounts. The experiments indicated that as the water addition amount increased, the volume shrinkage fluctuated minimally, while the density showed a significant decreasing trend. Sample W1 exhibited the lowest volume shrinkage and the highest density, approximately 0.16 g/cm^3^. This is primarily attributed to the slower hydrolysis–polycondensation reaction rate under low water addition, resulting in a higher cross-linking density of the siloxane network and the formation of a dense, uniform skeleton structure, which effectively inhibited skeleton deformation and shrinkage during drying. The volume shrinkage of sample W2 increased slightly, while those of W3 and W4 tended to stabilize. However, their densities decreased to 0.12 g/cm^3^ and 0.09 g/cm^3^, respectively. High water addition caused a decrease in the cross-linking density of the siloxane network, leading to a looser skeleton structure accompanied by increased porosity, ultimately resulting in a significant reduction in density.

#### 4.2.3. Thermal and Mechanical Characterization

Figure 9c shows the variation trend of thermal conductivity for samples W1–W4. As the water addition amount increased, the thermal conductivity of the aerogels showed a gradual decreasing trend. The thermal conductivity of W1 was 0.038 W/(m·K), while those of W2, W3, and W4 decreased to 0.030 W/(m·K), 0.034 W/(m·K), and 0.025 W/(m·K), corresponding to reductions of 20.1%, 10.7%, and 6.4%, respectively. A dense skeleton structure led to an increased thermal conductivity by enhancing solid-phase heat conduction pathways. Conversely, the loosened siloxane network structure results in significantly increased porosity, which effectively inhibits solid-phase heat conduction and lowers the overall thermal conductivity. When the water addition amount was increased to 7 mL (W4), the thermal conductivity reached its minimum value of 0.025 W/(m·K). At this point, the cross-linking density of the siloxane network was the lowest, forming a high-porosity structure; however, excessive water addition may lead to a reduction in skeleton strength and structural stability.

Previous analysis indicated that sample W1 had surface cracks and uneven morphology, while sample W4 exhibited poor optical performance, making both unsuitable as subjects for mechanical property testing. Therefore, this section selected samples W2 and W3 for mechanical characterization. Figure 9d shows their compressive strain–stress curves and resilience characteristics. It can be observed that the compressive strength of the W3 aerogel was significantly lower than that of W2, and its deformation recovery capability was also somewhat reduced. The compressive strength of W2 reached 0.59 MPa, whereas that of W3 was only 0.34 MPa, indicating that increased water addition led to a looser silicon skeleton structure. It is noteworthy that neither W2 nor W3 exhibited brittle unloading characteristics during compression, and the materials maintained excellent flexibility. A comparison of the recovery curves after 25% volume compression shows that although the resilience of W3 was slightly inferior to that of W2, the difference between them was relatively small, reflecting that the materials still possessed good reversible deformation characteristics within the regulated range of water addition.

#### 4.2.4. Summary

**Figure 10:**
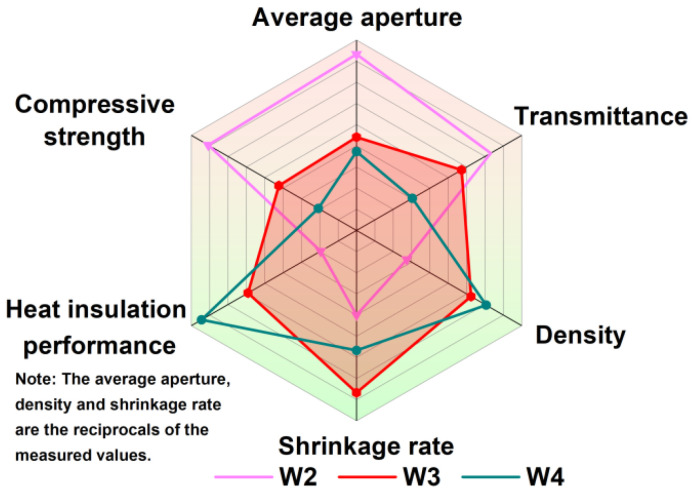
Comparison of the characterization parameters of samples W2–W4, normalized by the maximum value of each characteristic.

Figure 10 compares the various parameters of aerogel samples with different water addition amounts. Among them, although W3 was not superior in every individual property, it retained acceptable transmittance and flexibility while providing relatively low density and favorable thermal insulation. Therefore, 6 mL water addition was selected for subsequent optimization.

### 4.3. Effect of F127 Addition on the Properties of MTMS–DMDMS Aerogels

In the sol–gel process, F127, with its amphiphilic structure, stabilized the surface of siloxane particles, regulated the polycondensation rate, and inhibited network collapse. Simultaneously, the template structure formed by F127 self-assembly could effectively regulate the pore size distribution and improve the uniformity of the skeleton structure. Based on fixed parameters of *n*(MTMS:DMDMS) = 9:1 and a water addition amount of 6 mL, the effect of the F127 addition amount on aerogel properties was further investigated.

#### 4.3.1. Macroscopic Morphology

**Figure 11:**
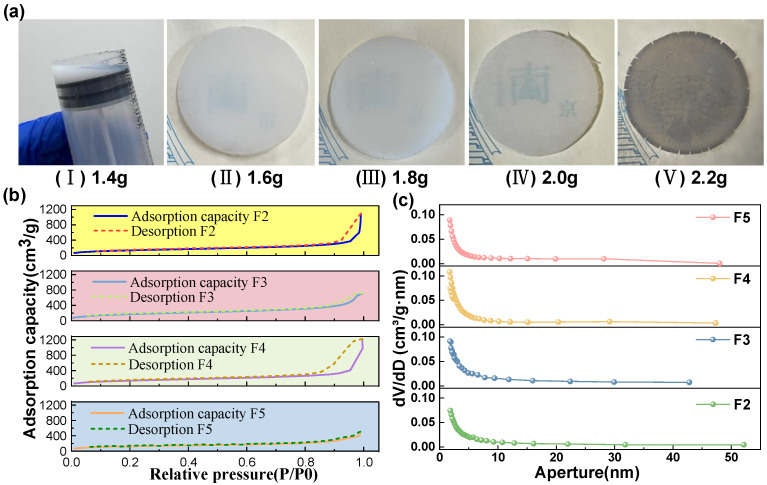
(**a**) Aerogels with different F127 additions. (**b**) N_2_ adsorption–desorption isotherms of aerogel samples F2–F5. (**c**) Pore size distribution of aerogel samples F2–F5.

Figure 11a shows the macroscopic morphology of SiO_2_ aerogels prepared under different F127 addition amounts, where (I)–(V) correspond to samples F1–F5, with F127 addition amounts of 1.4 g, 1.6 g, 1.8 g, 2.0 g, and 2.2 g, respectively.

Sample F1 failed to achieve effective stabilization due to insufficient F127 addition, leading to interface instability that terminated the polycondensation reaction, resulting in an ungelled state. Sample F2 exhibited some transparency but fragmented during drying with surface cracks. In contrast, samples F3 and F4 showed good transparency and integrity, indicating that within this addition range, F127 effectively coated the siloxane particle surfaces, regulating the polycondensation rate to form a dense and uniform siloxane network. The introduction of an appropriate amount of F127 inhibited skeleton collapse and mitigated capillary forces, giving the aerogel a smooth and transparent macroscopic appearance. However, when the F127 addition was increased to 2.2 g (sample F5), local inhomogeneity in the skeleton structure led to large defect regions within the pores. This caused stress concentration during drying, resulting in crack formation, and the sample became almost completely opaque.

In summary, the optimal addition range for F127 was 1.8 g to 2.0 g (corresponding to samples F3 and F4). Under this formulation, the aerogels exhibited excellent transparency, structural stability, and uniformity.

Table 5 presents the physical properties of aerogel samples F2–F5. It could be observed that with increasing F127 addition, the specific surface area and pore volume of the aerogels showed fluctuating changes. Sample F2 had relatively large and uniformly distributed pores, but a slightly lower specific surface area. Sample F3 exhibited a balanced performance in terms of moderate specific surface area and pore volume, along with uniform pore distribution. Sample F4 showed the largest pore volume and a relatively large average pore diameter, meeting the requirements for thermal insulation applications. Both the specific surface area and pore volume of sample F5 decreased to lower levels, indicating that excessive F127 may cause the collapse of the siloxane network structure, leading to uneven pore distribution and a weakening of the overall material performance.

Figure 11b shows the nitrogen adsorption–desorption isotherms of aerogel samples F2–F5. All samples exhibited characteristics of Type IV isotherms: in the low relative pressure region (P/P_0_ < 0.2), the adsorption quantity was low, indicating a minor contribution from micropores. The adsorption increased gradually with pressure, reflecting the uniformity of the pore structure. Within the medium relative pressure range (0.2 < P/P_0_ < 0.8), the adsorption quantity showed differentiated capillary condensation features with varying F127 addition. Notably, samples F3 and F4 exhibited a significant increase in adsorption, indicating a more developed mesoporous structure. In the high relative pressure region (P/P_0_ > 0.9), the adsorption increased sharply accompanied by distinct hysteresis loops, and the proportion of macropores varied with the F127 addition. It is noteworthy that sample F4 showed a sudden jump in adsorption and the widest hysteresis loop, revealing its largest pore volume and broadest pore size distribution, corresponding to the loosest structure. In contrast, sample F5 displayed significantly reduced adsorption and a narrower hysteresis loop, suggesting a densified internal structure with markedly decreased pore volume.

Figure 11c shows the pore size distribution characteristics of aerogels F2–F5. The results show that the pores of sample F2 were mainly distributed in the 10–15 nm range, presenting a typical mesoporous structure with uniform distribution. The pore size of sample F3 significantly decreased and concentrated around 7.25 nm, indicating that an appropriate amount of F127 refined the pores by regulating the network structure, forming a more concentrated mesoporous system. The pore size of sample F4 expanded significantly, showing obvious characteristics of a loosened skeleton structure with coexisting mesopores and some macropores. Sample F5 had the smallest pore size, approximately 6.08 nm, with a narrow distribution, suggesting that excessive F127 restricted pore development and induced localized collapse of the skeleton structure.

Comprehensive performance analysis indicates that if thermal insulation performance is the priority, sample F4 holds an advantage due to its larger pore volume and broader pore size distribution. However, when a balance among mechanical properties, adsorption performance, and thermal insulation is required, sample F3 emerges as the superior choice owing to its uniform pore size distribution and stable structure.

#### 4.3.2. Optical Characterization

**Figure 12:**
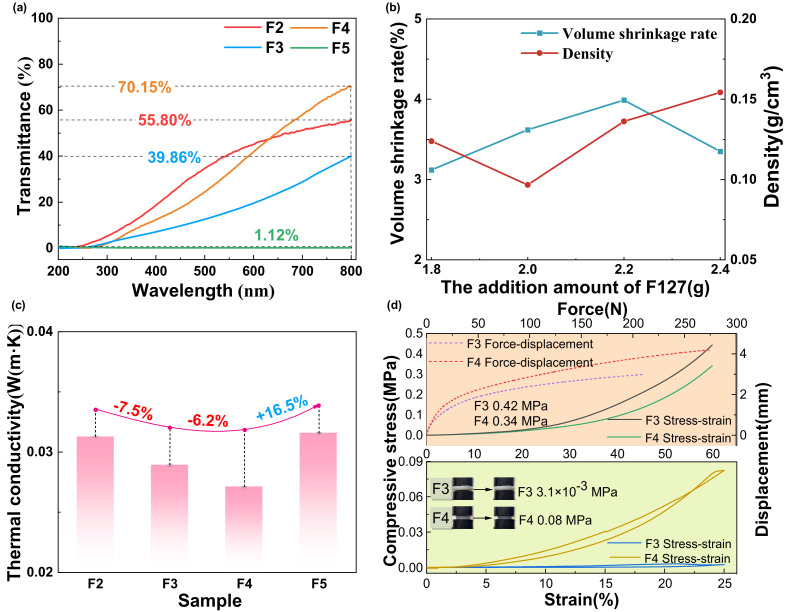
(**a**) Visible light transmittance of aerogel samples F2–F5. (**b**) Volume shrinkage ratio and density of aerogel samples F2–F5. (**c**) Thermal conductivity of aerogel samples F2–F5. (**d**) Stress–strain and compression resilience curves of aerogel samples F3 and F4.

Figure 12a shows the influence of F127 addition amount on visible light transmittance. As the F127 addition amount increased from 1.6 g to 2.0 g (corresponding to samples F2 to F4), the visible light transmittance of the aerogel showed a trend of first decreasing and then increasing. The transmittance values for samples F2, F3, F4, and F5 were 55.80%, 39.86%, 70.15%, and 1.12%, respectively. It is noteworthy that an appropriate amount of F127 regulated the pore structure to form a dense and uniform siloxane network, effectively shortening the light scattering path, which allowed sample F3 to achieve the highest transparency. When the F127 addition amount exceeded 2.0 g (samples F4–F5), the residues introduced during its formation interfered with the cross-linking of the siloxane network, leading to irregular pore structures and consequently a significant reduction in transparency.

Figure 12b shows that with increasing F127 addition, the volume shrinkage and density of the aerogels exhibited fluctuating trends. When the F127 addition reached 2.2 g, the volume shrinkage peaked, indicating that an appropriate amount of F127 could effectively inhibit skeleton collapse during drying by enhancing the stability of the pore structure. However, when the addition exceeded 2.0 g, the volume shrinkage slightly decreased instead, suggesting that excessive surfactant weakened the skeleton’s flexibility and resistance to shrinkage. The density trend showed an inverse relationship with volume shrinkage, reaching its minimum at an F127 addition of 2.0 g. This originated from the enhanced surface activity regulation by a higher F127 content during the sol–gel process, promoting skeleton densification. The experimental data indicate that an F127 addition range of 1.8–2.0 g effectively balanced density and volume shrinkage, exhibiting optimal comprehensive performance. Although a higher addition (e.g., 2.4 g) reduced the volume shrinkage, it was accompanied by an increase in density, and its negative impacts on the aerogel’s transparency and flexibility still require consideration.

#### 4.3.3. Thermal and Mechanical Characterization

Figure 12c shows the variation pattern of thermal conductivity for samples F2–F5. The results indicate that with increasing F127 addition, the thermal conductivity of the aerogels exhibited a “U-shaped” variation trend, first decreasing and then increasing. The thermal conductivity of samples F2–F4 decreased from 0.031 W/(m·K) to 0.027 W/(m·K). However, when the addition amount was increased to 2.2 g, the thermal conductivity rebounded. This is attributed to the excessive F127 residue within the skeleton enhancing the solid-phase heat conduction pathways. An appropriate amount of F127 can significantly improve thermal insulation performance by optimizing the network structure distribution, whereas the residue introduced by excessive addition disrupts the original thermal resistance structure and increases thermal conductivity. Comprehensive performance analysis shows that adding 2.0 g of F127 maintains a low thermal conductivity of 0.027 W/(m·K) while balancing mechanical and optical properties, demonstrating optimal thermal insulation performance.

Based on the comprehensive analysis of macroscopic morphology and optical performance, samples F2 and F5 were excluded due to insufficient transparency, and samples F3 and F4 were selected for mechanical property testing. Figure 12d shows the compressive strain–stress curves and resilience characteristics of the F3 and F4 aerogels. The experiments demonstrated that the maximum compressive strength of sample F3 reached 1.24 MPa, while that of sample F4 was only 0.34 MPa. The increase in F127 addition significantly reduced the load-bearing capacity of the material, which may originate from its regulatory effect on the cross-linking degree of the siloxane network, leading to an overall looser structure. The compression–recovery curves show that sample F3 exhibited a distinct fracture point after 30% compressive deformation, indicating irreversible damage to the skeleton structure under stress and a sharp subsequent decline in recovery ability. In contrast, sample F4 showed a smooth recovery curve, reflecting better structural integrity and elastic recovery.

#### 4.3.4. Summary

**Figure 13:**
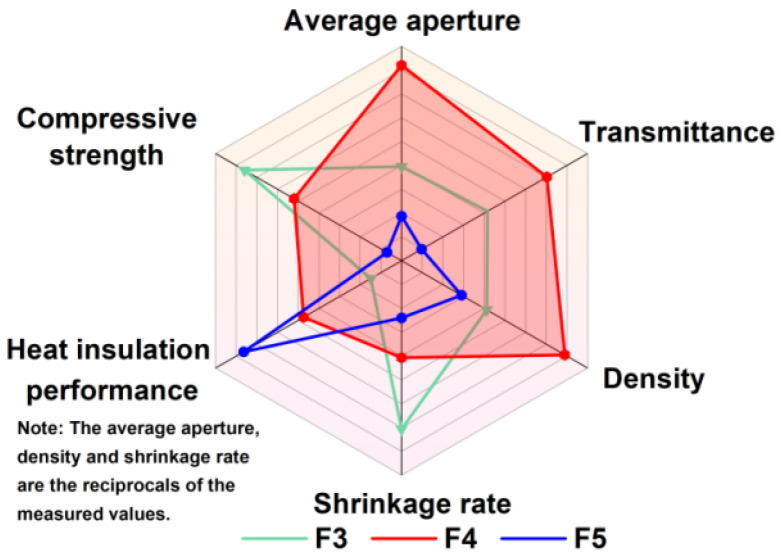
Comparison of the characterization parameters of samples F3–F5, normalized by the maximum value of each characteristic.

Figure 13 compares the parameters of aerogel samples with varying F127 additions. The radar plot shows that sample F4 exhibited the largest pore size and the lowest thermal conductivity, while maintaining acceptable mechanical recovery and optical performance. In contrast, the insulation performance of sample F3 is inferior, and its transmittance is also not outstanding. Sample F5 performs poorly in all aspects other than the insulation property. Based on the research objectives, 2.0 g of F127 was selected.

## 5. Conclusions

This study employed an acid-base two-step catalysis, sol–gel method, and ambient pressure drying technology to prepare SiO_2_ aerogels using MTMS as the silicon source. It systematically investigated the effects of the type of alkaline catalyst, water-to-MTMS ratio, and surfactant on the gelation mechanism, microstructure, and optical properties. Building upon this, DMDMS was introduced to improve material flexibility, and the composite silicon source ratio and process parameters were optimized. The main conclusions are as follows:

(1) TMAOH demonstrates excellent performance in regulating the reaction rate, optimizing the network structure, and enhancing transparency. Its optimal addition amount is *n*(TMAOH):*n*(MTMS) = 0.071:1. When the water-to-MTMS ratio is *n*(H_2_O):*n*(MTMS) = 7:1, a dynamic balance between hydrolysis and polycondensation is achieved, forming a gel network with uniform structure and stable skeleton, which effectively inhibits damage from capillary forces during the drying stage. Using F127 as the surfactant enables the construction of a fine skeleton with a fibrous, branched structure, significantly improving the uniformity of the pore size distribution.

(2) MTMS is rich in hydrophobic groups. MTMS-based SiO_2_ aerogels exhibit relatively intact macroscopic morphology, excellent transparency, hydrophobicity, and low thermal conductivity. The experimental results show that the SiO_2_ aerogel formed with TMAOH as the alkaline catalyst, a water/MTMS ratio of 7:1, and F127 as the surfactant exhibits optimal performance. Its density is 0.18 g/cm^3^, with high uniformity in pore size distribution, a porosity of 93.50%, a water contact angle of 125.98°, and a transmittance as high as 89.74%.

(3) The introduction of DMDMS significantly improves the material’s flexibility and crack resistance, increasing the compressive strength by 22.4% compared to the pure MTMS system. When the MTMS/DMDMS volume ratio is 9:1, the water addition amount is 6 mL, and the F127 addition amount is 2.0 g, the resulting composite aerogel achieves synergistic optimization of mechanical, optical, and thermal properties while maintaining a transmittance of 70.15%, demonstrating its potential for application in the field of transparent thermal insulation materials.

Furthermore, the as-prepared SiO_2_ aerogels demonstrate favorable potential for practical engineering applications. The ambient pressure drying route employed in this work is favorable for scaled-up production due to low equipment demand, mild preparation conditions, and low energy consumption. In addition, the resultant aerogels possess good structural stability and can maintain intact morphology, satisfactory transparency, and stable performance during long-term storage for more than 3 months without obvious degradation, which is critical for practical applications in transparent thermal insulation fields.

## Figures and Tables

**Table 1:** Experimental materials and reagents.

Reagent Name	Purity	Manufacturer
TEOS	99.0%, AR	Aladdin Biochemical Technology Co., Ltd. (Shanghai, China)
MTMS	98.0%, AR	Aladdin Biochemical Technology Co., Ltd.
DMDMS	99.9%, AR	Aladdin Biochemical Technology Co., Ltd.
Acetic acid (CH_3_COOH)	99.5%, AR	Sinopharm Chemical Reagent Co., Ltd. (Shanghai, China)
Aqueous ammonia (NH_3_·H_2_O)	25.0%, AR	Sinopharm Chemical Reagent Co., Ltd.
Urea	99.0%, AR	Aladdin Biochemical Technology Co., Ltd.
TMAOH	99.0%, AR	Aladdin Biochemical Technology Co., Ltd.
CTAB	99.0%, AR	Aladdin Biochemical Technology Co., Ltd.
Pluronic F-127	Average Mn~12,600	Aladdin Biochemical Technology Co., Ltd.

**Table 2:** Detailed information of experimental instruments.

Instrument	Manufacturer	Model	Accuracy	Others
Heating magnetic stirrer (Two-unit)	KEMS-GSS (Bengaluru, India)	Nanjing Cole	——	Rotational speed range: 0~1500 rpm
Vacuum drying Oven	Shanghai Jiecheng (Shanghai, China)	DZF-6020	Temperature: ±1 °C	Used for drying
Electronic balance	PMK224ZH (Shanghai, China)	OHAUS	Precision: ±0.1 mg	——
Thermal conductivity meter	SwedenHot Disk (Gothenburg, Sweden)	TPS2500S	0.005–500 W/(m·K)	Used for measuring thermal conductivity
Contact angle measuring instrument	Kunshan Shengding (Suzhou, China)	SDC 350KS	±0.1°	Used for measuring the hydrophobic angle
Specific surface area and porosity analyzer	The U.S.A Micromeritics (Norcross, GA, USA)	ASAP 2460	——	Used for aperture analysis
Visible diffuse reflection test	Japan Shimadzu (Kyoto, Japan)	UV-3600	Wavelength: ±1.0 nm	Used for optical testing
SEM	Germany ZEISS (Oberkochen, Germany)	Sigma 300	——	Used for observing microstructure

**Table 3:** Physical properties of aerogel samples S3–S5.

Sample Number	Specific Surface Area (m^2^/g)	Pore Volume (cm^3^/g)	Average Pore Size (nm)
S3	592.62	0.72	6.08
S4	579.77	1.13	9.24
S5	574.16	3.31	23.21

**Table 4:** Physical properties of aerogel samples W1–W4.

Sample Number	Specific Surface Area (m^2^/g)	Pore Volume (cm^3^/g)	Average Pore Size (nm)
W1	548.45	2.47	18.83
W2	517.05	2.56	20.53
W3	574.16	3.31	23.21
W4	668.92	3.83	23.71

**Table 5:** Physical properties of aerogel samples F2–F5.

Sample Number	Specific Surface Area (m^2^/g)	Pore Volume (cm^3^/g)	Average Pore Size (nm)
F2	503.36	1.59	13.48
F3	616.31	0.96	7.25
F4	574.16	3.31	23.21
F5	446.94	0.71	6.08

## Data Availability

The original contributions presented in this study are included in the article. Further inquiries can be directed to the corresponding authors.

## Abbreviations

The following abbreviations are used in this manuscript:

*Nomenclature*	
*m*	Sample mass, g
*L*	Thickness, mm
*P*	Porosity, %
*V*	Sample volume, cm^3^
*Greek symbols*	
*ρ*_1_	Silicon skeleton density, g/cm^3^
*ρ*	Apparent density, g/cm^3^
*λ*	Wavelength, nm
*Acronyms*	
CTAB	Cetyltrimethylammonium bromide
DMDMS	Dimethyl dimethoxy silicane
F127	Pluronic F-127
MTES	Methyltriethoxysilane
MTMS	Methyltrimethoxysilane
POSS	Polyhedral oligomeric silsesquioxane
SEM	Scanning electron microscope
TEOS	Tetraethyl orthosilicate
TMAOH	Tetramethylammonium hydroxide
TMOS	Tetramethyl orthosilicate
